# Supplementary material for: Mechanisms governing the pioneering and redistribution capabilities of the non-classical pioneer PU.1
Source: Nat Commun. 2020 Jan 21;11:402. doi: 10.1038/s41467-019-13960-2 (PMC6972792; doi:10.1038/s41467-019-13960-2)
Supplement: Supplementary file 7 — Source data [file 41467_2019_13960_MOESM7_ESM.zip › Source_Data/FigureS2/FigureS2B_MotifScanOutput/KmeansCluster6/homerResults/motif10.similar.html]

motif10

## Information for motif10

T
C
A
G
C
G
A
T
T
C
A
G
T
G
C
A
A
G
C
T
C
G
T
A
A
T
C
G
C
G
A
T
T
C
A
G
C
T
G
A
A
T
C
G
A
C
G
T
  
Reverse Opposite:  

T
G
C
A
T
A
G
C
G
A
C
T
A
G
T
C
G
C
T
A
T
A
G
C
G
C
A
T
T
C
G
A
A
C
G
T
A
G
T
C
G
C
T
A
A
G
T
C
  

|  |  |
| --- | --- |
| p-value: | 1e-70 |
| log p-value: | -1.613e+02 |
| Information Content per bp: | 1.708 |
| Number of Target Sequences with motif | 152.0 |
| Percentage of Target Sequences with motif | 4.62% |
| Number of Background Sequences with motif | 245.1 |
| Percentage of Background Sequences with motif | 0.57% |
| Average Position of motif in Targets | 102.7 +/- 55.5bp |
| Average Position of motif in Background | 103.0 +/- 71.9bp |
| Strand Bias (log2 ratio + to - strand density) | -0.1 |
| Multiplicity (# of sites on avg that occur together) | 1.01 |
| Motif File: | file (matrix) reverse opposite |

### Similar de novo motifs found

|  |  |  |  |  |  |  |  |
| --- | --- | --- | --- | --- | --- | --- | --- |
| Rank | Match Score | Redundant Motif | P-value | log P-value | % of Targets | % of Background | Motif file |
| 1 | 0.965 | C A G T T C A G G C T A A G C T C G T A T A C G A G C T T C A G C G T A A C T G A G C T | 1e-64 | -148.731195 | 3.31% | 0.26% | motif file (matrix) |
| 2 | 0.919 | A T C G C G T A A C G T C G T A C T A G A G C T C T A G C G T A A C T G A C G T | 1e-52 | -121.273481 | 2.92% | 0.27% | motif file (matrix) |
| 3 | 0.817 | T C G A G C T A T G C A A T C G G A C T C T A G G T C A T A C G A C G T | 1e-37 | -87.454576 | 3.98% | 0.89% | motif file (matrix) |
| 4 | 0.815 | C G T A A C T G A C G T A C T G C G T A A C T G A C G T | 1e-28 | -65.789075 | 8.35% | 3.83% | motif file (matrix) |
| 5 | 0.831 | C G T A A G T C A C G T A G T C C G T A A G T C A C G T C G T A | 1e-28 | -65.738281 | 3.92% | 1.12% | motif file (matrix) |
